# A Unified View of “How Allostery Works”

**DOI:** 10.1371/journal.pcbi.1003394

**Published:** 2014-02-06

**Authors:** Chung-Jung Tsai, Ruth Nussinov

**Affiliations:** 1 Cancer and Inflammation Program, Leidos Biomedical Research, Inc., Frederick National Laboratory, National Cancer Institute, Center for Cancer Research, Frederick, Maryland, United States of America; 2 Sackler Institute of Molecular Medicine, Department of Human Genetics and Molecular Medicine, Sackler School of Medicine, Tel Aviv University, Tel Aviv, Israel; UNC Charlotte, United States of America

## Abstract

The question of *how allostery works* was posed almost 50 years ago. Since then it has been the focus of much effort. This is for two reasons: first, the intellectual curiosity of basic science and the desire to understand fundamental phenomena, and second, its vast practical importance. Allostery is at play in all processes in the living cell, and increasingly in drug discovery. Many models have been successfully formulated, and are able to describe allostery even in the absence of a detailed structural mechanism. However, conceptual schemes designed to qualitatively explain allosteric mechanisms usually lack a quantitative mathematical model, and are unable to link its thermodynamic and structural foundations. This hampers insight into oncogenic mutations in cancer progression and biased agonists' actions. Here, we describe how allostery works from three different standpoints: thermodynamics, free energy landscape of population shift, and structure; all with exactly the same allosteric descriptors. This results in a unified view which not only clarifies the elusive allosteric mechanism but also provides structural grasp of agonist-mediated signaling pathways, and guides allosteric drug discovery. Of note, the unified view reasons that allosteric coupling (or communication) does not determine the allosteric efficacy; however, a communication channel is what makes potential binding sites allosteric.

In cell biology the ability to perform a biological function is determined by how populated a macromolecule in its active conformation is. Rather than direct manipulation of the active (functional) site, allostery is capable of altering the active state population by some perturbation away from the active site, such as that elicited by ligand binding, post-translational modifications (PTMs), and more [1]. Nature has exploited allosteric regulation in the cellular network for signal transduction [2], enzyme activation [3], metabolism regulation [4], motor work [5], and transcription control [6], [7]. To perform cellular functions via allostery, evolution has optimized multiple free energy basins at the bottom of the folding funnel [8], [9]. As illustrated in Figure 1, in a typical free energy landscape [10] allosteric activation operates as a robust bi-stable switch through a narrow window of allosteric ligand concentration, shifting the population from the inactive to the active state. Such concentration-dependent behavior is taken for granted and only gets noticed when homeostasis is broken, through constitutive activation by mutations. Not surprisingly, the important role of allostery in cellular circuits, spelled by genetic sequences blueprints, has been recognized as “the second secret of life,” second only to the genetic code [11], [12].

**Figure 1:**
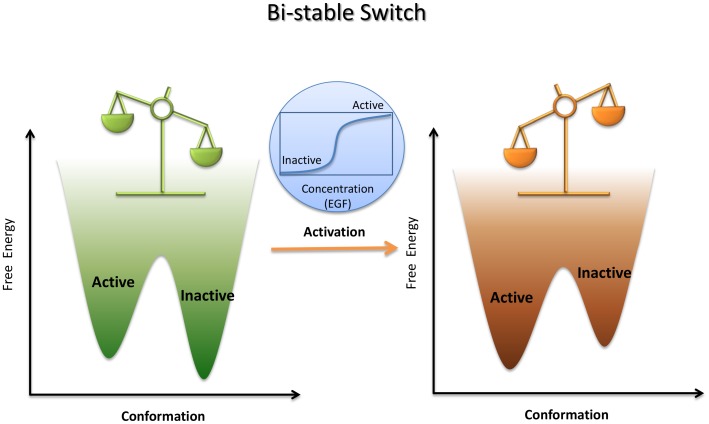
A typical allosteric activation via a bi-stable switch. A node in the cellular network is illustrated by only two populated states, active and inactive, separated by a sizeable but surmountable free energy barrier. Before activation, the inactive state dominates the population as indicated by the relative basin depth in the free energy landscape and a balance level. Within a narrow increment range in ligand concentration, the allosteric activation event shifts the population in favor of the active state. The activation is highlighted in the embedded plot with a typical sigmoid transition from the inactive to the active state.

Numerous approaches have been undertaken over the last 50 years in an effort to explain allostery and to study its underpinnings and consequences [13]. Each theme contributed to deepen and strengthen our grasp of the allosteric phenomena. However, until recently the principles did not intersect nor converge to provide a global picture [14], [15]. Broadly, studies of allostery fall into three mainstream categories. The first is based on the principle of thermodynamic equilibrium. Several mathematical models have been formulated, seeking a quantitative description with measurable allosteric properties. Even though they offered allosteric quantification, the thermodynamic outlook gave no indication as to why similar ligands bound at the same allosteric site may result in opposite agonism [16]. Second, conceptual models such as conformational selection versus induced fit were developed to explain the mechanism of how biological functions are achieved through allostery. Although in terms of the free energy landscape the conceptual thermodynamic view of conformational selection with population shift is directly linked to structural changes, to date no quantitative connection has been construed. The third embodies numerous implicit and explicit approaches which exploited the inferred structural coupling between the functional (active) and allosteric binding sites in a host protein. These implied that structural linkage is a necessary condition for an allosteric action. However, the structural view of allostery has been questioned since according to one of the thermodynamic models detailed structural information is not required [14]. Here, our first goal is to quantitatively link between experimental allosteric properties and the relative changes in energy between the distinct (active, inactive) conformational states in an allosteric switch. To accomplish this for the conceptual population shift between these two dominant states, we employ the simplest thermodynamic allosteric two-state model (Figure 1). The emerging linkage serves as a new fundamental basis for unravelling the structural mechanism of allostery. Simply put, the linkage indicates that an allosteric ligand binding event, whether acting as an agonist or an (inverse) antagonist, depends on specific interactions between the ligand and the host protein. Surprisingly, this simple reasoning of agonism is opposite to the ensemble allosteric model (EAM) [17] which postulated that agonism is robustly encoded in the ensemble and does not require different interactions between the ligand and the host. Next, we acknowledge that the structural coupling between the functional and allosteric sites of a host protein is indeed specified by a connecting allosteric propagation pathway. We reason that while the propagation pathway itself does not play a role in determining ligand agonism, it both specifies the relative populations (i.e., stabilities) of the conformational states before ligand binding and modulates the allosteric ligand efficacy. We then proceed to apply the established linkage which is based on a thermodynamic view to the structural view of allostery by assigning the quantitative allosteric measure to be the structural coupling factor between two communicating sites. The simplified structural description with one allosteric ligand and two conformational states then paves the way toward specifying the criteria of agonist classification for two allosteric ligands with two functional sites. We note that over the years the Karplus' group has carried out several dynamic analyses from these three angles, including on chaperonin GroEL [18], yeast chorismate mutase [19], and Myosin V [20].

The first realization of allostery in biology was the cooperative binding of O_2_ to tetrameric hemoglobin (Hb). Among the many models formulated to account for this cooperativity, the two-state concerted MWC model [21] put forth by Monod, Wyman, and Changeux in 1965, which with only three parameters adequately described allosteric cooperativity, stands out as the best model at that time. The lack of a structural mechanism in the MWC model was filled by Perutz in 1970 based on careful comparison between x-ray structures of liganded Hb and deoxy Hb [22]. In 1972, Szabo and Karplus presented an allosteric model of the statistical mechanics of cooperativity [23]. To formulate the dependence of the three parameters in the MWC model [21] on structure, this allosteric model incorporated structural information relating to the conformational change in the salt bridge as observed by Perutz, as well as proton concentration, as indicated by the pH-dependence of ligand binding (Bohr effect). More general models, which account for the allosteric cooperativity under various experimental conditions, were discussed in recent reviews [24]–[26].

Following the successful allosteric cooperative binding models, many conceptual allosteric schemes [27]–[29], statistical mechanics models [17], [30], and general allosteric models [31]–[34] have been developed either for understanding how the allosteric mechanism works or for describing the allosteric behavior quantitatively. To address pharmacological needs, operational models [32]–[34] were developed to measure the quantitative allosteric efficacy of drugs from experimental response-concentration curves. This ability to quantitatively measure allosteric efficacy makes the ***thermodynamic view of allostery*** a major foundation of allostery. In the diverse mathematical equations formulated for this purpose, each species included in the equation is only given one conformational state and the population is specified by its concentration. The limitations of the operational models, due to the omission of the concept of population shift in species responsible for pharmacological response, have been pointed out in a recent review [35].

In 1995, Leff presented the simplest two-state model [36] for receptor activation by an agonist, which is schematically identical to the MWC model but applied to monomers instead of oligomers. This simplest allosteric two-state model (ATSM) is depicted in Figure 2A with a slight difference from its original presentation. As indicated in the figure, the ATSM implicitly incorporated the schematic framework of conformational selection and population shift [9], [37]; however, this important concept of allostery has not been explicitly emphasized in formulating the quantitative allosteric behavior. Nonetheless, the best way to convey the concept of conformational selection and population shift is through a one-dimensional sketch of the free energy landscape in terms of the conformational space [8], [38]. In a typical free energy landscape representation, such as that shown in Figure 3, the number of minima corresponds to the number of dominant states and their populations are determined by the relative free energy depth in each basin. Given two overlapping free energy landscapes, respectively for the apo, which is favorable for the inactive state, and the complex, which is favorable for the active state, the allosteric effect elicited by ligand binding can be clearly visualized by population shift toward the favored active conformation. Such an energy landscape sketch represents the conceptual thermodynamic view of allostery. Below, we quantitatively label the energy landscape sketch with parameters derived from a thermodynamic mathematical model and link these parameters to the second main foundation, the structural view of allostery [39]. To our knowledge, this will be the first time that conceptual population shift through allosteric binding has been correlated with quantitative measurable parameters. A similar free energy landscape of activation of the signaling protein NtrC [8] has been correlated with microscopic rate constants between the inactive and active states, which were extracted from fitted global exchange rate constant (K_ex_) [40] using rate constants determined by the NMR ^15^N backbone amide CPMG (Carr-Purcell-Meiboom-Gill) relaxation dispersion experiments [41]. The application of NMR techniques to the study of allostery has been described for several examples in a recent review [42].

**Figure 2:**
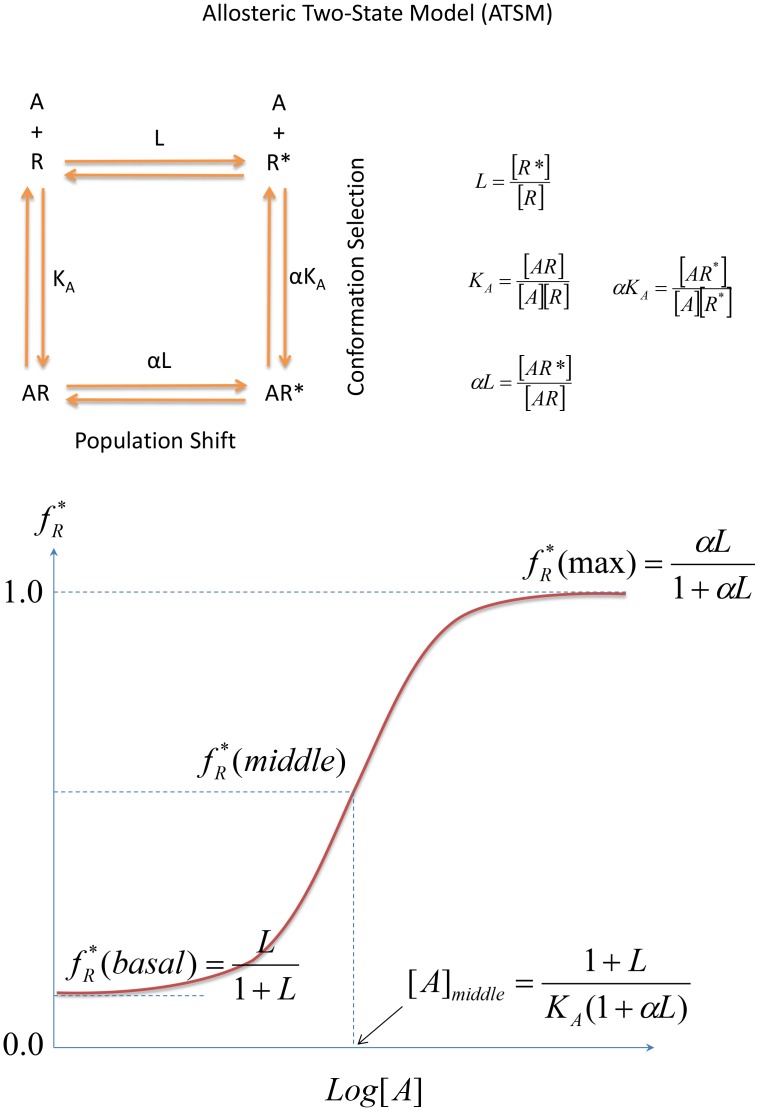
The simplest allosteric two-state model (ATSM). (A) The two-state model presents an equilibrium between two states, 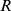 and 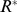, with the relative population defined by the equilibrium constant, 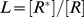, and their binding to an allosteric ligand, 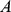. For the inactive state, the binding equilibrium constant is given by 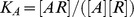, and for the active state, by 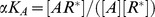. Due to the complete circle of equilibrium, the equilibrium constant between 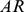 and 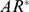 is automatically deduced as 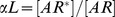 with the previous three mass equations. Also, the forward reaction 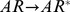 with 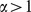 implies a population shift due to the allosteric binding event. In this schematic allostery description, the conformation selection scheme emphasizes that the microscopic path of 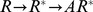 dominates the equilibrium process in contrast to the induced-fit scheme which implies the 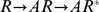 path prevails. (B) A typical sigmoid response-concentration curve in the allosteric two-state model. If we accept the assumption that a measured biological response is proportional to the fraction of receptors in the activated state, 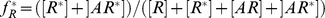 as defined in the ATSM, manipulation of the three equilibrium equations in ATSM (Figure 2A) deduces the response, 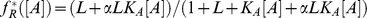, as a function of ligand concentration with three independent parameters, 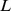, 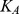, and 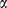. The sigmoid response-concentration curve of ATSM is established by three quantities, the basal activity as 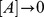, 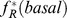, the maximum activity 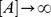, 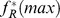, and the activity at the middle point of the transition, 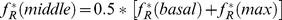 which corresponds to ligand concentration at 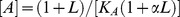.

**Figure 3:**
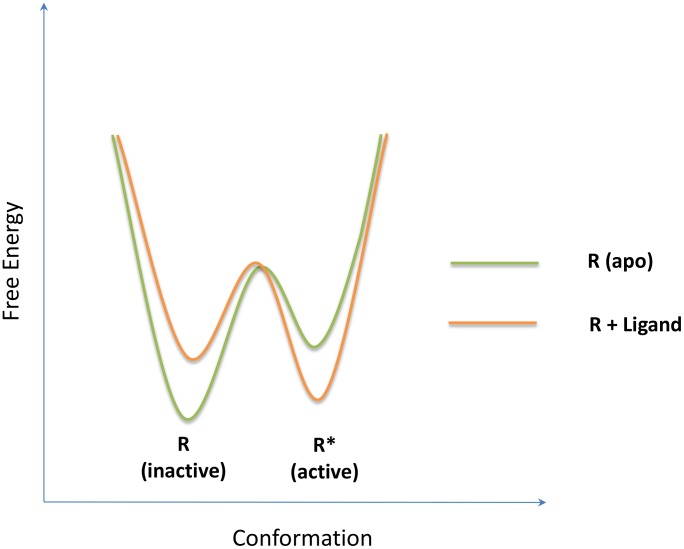
The simplest free energy landscape presentation of the thermodynamic view of allostery. At the bottom of the folding funnel, an apo protein is optimized to populate two states, 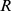 (inactive) and 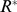 (active), with each basin representing an ensemble of conformations and their relative populations as determined by the relative depth of the local basins. Allostery is clearly seen by a population shift from the inactive state dominated by apo (light green) to the active state prevailing in the complex (pale orange) through allosteric ligand binding.

The ***structural view of allostery*** considers the interactions among a set of residues as responsible for the allosteric coupling between the allosteric and functional sites. It reasons that allosteric communication is specified by allosteric networks (or channels) through which strain energy created at the allosteric site by binding, PTM, or mutations propagates to the functional site and induces a conformational change [43]. This propagation view corresponds well to the intuitive induced-fit description of allostery. It is supported by a sequence-based statistical method illustrating a connection between two sites through inferred allosteric networks [44]. Many theoretical studies explored a variety of distinct methods to reveal hidden allosteric communications, including molecular dynamics [45]–[47], path enumeration [48], evolutionary trace analysis [49], atomistically detailed minimum energy path [50], and statistical structural analysis [51], [52], as well as covariance analysis of NMR chemical shifts [53].

At first glimpse, these two—thermodynamic and structural—allosteric views do not overlap much except for the end result of allosteric activation, and neither view requires the other to explain allostery. However, the free energy landscape allows a unified view which emphasizes the complementarity rather than the contradiction. Starting from the simplest allosteric two-state model, we tag the population shift in the free energy landscape with quantitative allosteric couplings which can be derived from an experimental response-concentration curve. To forge a tight link to the thermodynamic view of allostery, we then use the same descriptors for the structural view. Because common descriptors are adopted, the unified framework (1) illuminates how allostery works; (2) helps in accurately classifying agonist types observed in the literature based on quantities of allosteric efficacy determined by experiments; and most importantly, (3) provides the basis for unraveling the structural mechanism of allostery.

Below, we first clarify the terminology of orthosteric agonist, which has been defined as a naturally occurring endogenous ligand of G protein–coupled receptors (GPCRs) [54]. To emphasize the allosteric effect, we and many others consider a ligand bound at the active or functional site an orthosteric ligand or agonist. This discrepancy in definition results from the fact that an orthosteric agonist bound close to the extracellular part of GPCR is by default an allosteric ligand, since the activation or regulation site is located in the intracellular part of GPCR, while the term allosteric agonist is reserved for ligands bound at sites other than the “orthosteric” site of GPCR, functioning as modulator to the orthosteric agonist. Thus, it is important to clarify that in our description of allostery both “orthosteric” agonists and “allosteric” ligands bound to GPCR are allosteric ligands.

## Allosteric Two-State Model (ATSM)

The functional efficacy of a protein depends on how it populates its active conformation. As a node in the cellular circuit, a protein is expected to be able to switch its functional mode between on/off states. To fulfil its biological role, a protein has been optimized by evolution not only to populate a single active conformation; instead, two or more switchable states including the folded or disordered state need to be populated as well. Each conformation (or state) corresponds to a local free energy minimum at the bottom of the folding funnel. If the triggering event that effectively switches the protein population from one state to the other is far away from the active site of the protein, as for example in the case of substrate binding or covalent modification (PTM) distal to the functional site, it is referred to as allostery and the trigger site is termed an allosteric site. Since allostery plays significant roles in the cell, two distinct but compensatory approaches have been developed to explain how allostery works: one from a thermodynamic viewpoint, the other from a structural standpoint.

The thermodynamic view focuses on how to accurately describe allosteric phenomena, such as the classical cooperative oxygen binding to haemoglobin, without involving detailed structural information. The mathematical models below are mainly based on simple thermodynamic principles. On the other hand, the structural view emphasizes that optimized allosteric communication (or coupling) between allosteric and active sites is responsible for the allosteric conformational switch. It envisages that strain energy, created at an allosteric site by a triggering event, will propagate to the active site via an allosteric coupling channel to alter the functional conformation. The sequence-based statistical method for estimating thermodynamic coupling between residues in proteins has successfully visualized allosteric coupling in long-range energetic interactions for three proteins [44]. This concept has also inspired the discovery of allosteric binding sites and mutations via correlated movements from trajectories obtained from molecular dynamics simulations [55].

The simplest yet practical model for the thermodynamic view is the two-state model [36]. As defined in Figure 2A, a protein (or receptor) can populate one of two states: the inactive 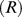 or active 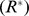. In the free form, their distributions are governed by the equilibrium constant 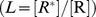. According to the conformational selection concept, a ligand (*A*) will preferentially bind one state over the other. The binding affinities are quantitatively defined by association constants, with 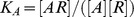 for an inactive and 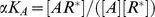 for an active conformation. The thermodynamic relationships in Figure 2A indicate that the binding affinity ratio of ligand 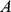 for 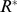 and 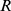, 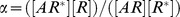, becomes the allosteric intrinsic efficacy of ligand 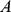 with 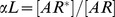. The population shift toward the active state following binding is proportional to the fraction of receptors in the activated state, 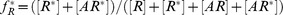. Manipulation of the ATSM equations given in Figure 2A works out the 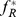 as a function of ligand concentration 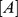, with three independent parameters, 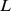, 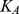, and 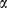, giving 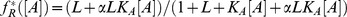. The basal activity 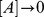 is reduced to 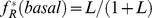; the maximum activity 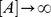 is given as 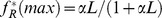; and the activity at the middle point of the transition, 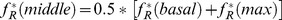, corresponds to a ligand concentration of 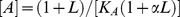. The summation of ATSM in Figure 2B shows the characteristic sigmoid activation curve from basal activity through a middle point transition to maximum activity.

**Figure 4:**
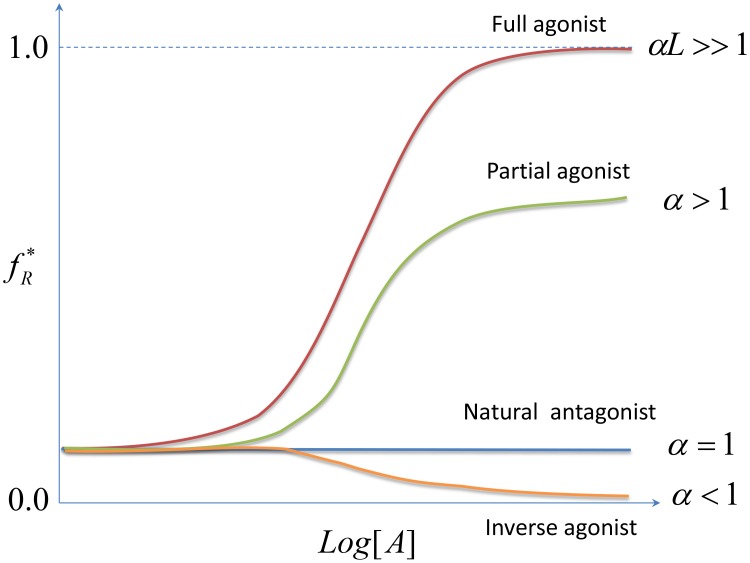
The classification of allosteric ligands with ATSM. Given an experimental sigmoid response-concentration curve with full biological response, we can determine the three independent parameters 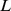, 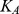, and 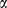 in ATSM. Full agonist, corresponding to 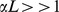, produces a full biological response. Partial agonist even at saturating concentration can only produce a partial biological response with 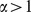. Inverse agonist suppresses basal activity with 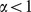. Neutral antagonist with 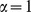 does not impose any biological response.

The ATSM offers allosteric ligands a functional classification through the measured allosteric intrinsic efficacy, *α*. As indicated in Figure 4, a full agonist can reach nearly 100% activity with 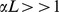. A partial agonist corresponds to 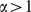. A natural antagonist is defined by 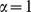 where the ligand shows no binding preference, in contrast to an inverse agonist 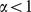 where the ligand preferentially binds the inactive state conformation. The two conceptual allosteric frameworks, conformational selection and population shift, fuse through the same parameter *α* in the ATSM, however with differing descriptions, binding affinity, and allosteric intrinsic efficacy. Nonetheless, despite the simple yet powerful description, the ATSM is unable to provide a direct connection between structure and function. To link the thermodynamic view with the structural view, we next sketch the relative free energy between the two states in the ATSM.

## Free Energy Landscape with Allosteric Efficacy, *α*

**Figure 5:**
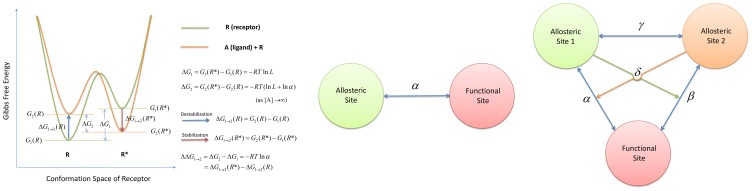
The thermodynamic and free energy landscape of the population shift views, the structural view of the allosteric two-state model, and an extension of the model to two allosteric sites and one functional site. (A) The free energy landscape presentation of ATSM. Before binding, the relative free energy between the inactive (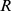) and active (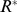) states is given by 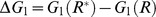, which is 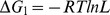 according to the ATSM as depicted by the light green curve. After binding, the relative free energy between 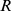 and 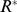 is given by 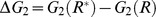, which under a saturating ligand concentration becomes 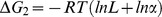, as drawn by the orange curve. The extent of population shift as measured by the free energy change due to binding, 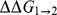, is equal to 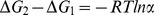. This result implies that the allosteric effect is solely determined by the allosteric efficacy, *α*, but not the absolute ligand affinity. 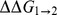 can also be expressed by the difference between the active conformation ***stabilization energy***, 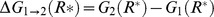 (red arrow), and inactive conformation ***destabilization energy***, 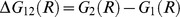 (blue arrow). (B) The structural view of allostery according to the ATSM. The allosteric communication between the allosteric and functional sites is indicated by the arrow with the coupling specified by the allosteric efficacy 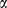. Unlike the thermodynamic view, the structural view emphasizes that the conformations of two sites breathe dynamically in a concerted motion through a set of mutually interacting residues. Without such a propagation channel between sites, 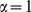 is always the case, no matter the changes at the allosteric site. Thus, while a preexisting channel (or allosteric networks of correlated residues) is a required condition, by itself the communication through the channel does not determine the allosteric efficacy. (C) The structural view of allostery according to the extended ATSM. In the drawing, the two allosteric communication channels between the two allosteric sites and the functional site are indicated by the blue double arrows with the coupling specified by the allosteric efficacy 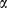, 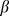 from the extended ATSM. The communication between the two allosteric sites is linked with a coupling specified by the binding cooperativity, 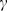, which is shown not to affect the allosteric efficacy directly. The activation cooperativity 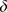 is the sum of the allosteric effect of site 1 toward coupling 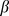 (pale green arrow) plus allosteric site 2 toward allosteric coupling *α* (orange arrow). As in the simplest ATSM, it is the ligand binding itself that puts forth the allosteric communications through existing propagation channels and determines the allosteric efficacy and the activation cooperativity either positively or negatively.

The one-dimensional free energy landscape [10] provides a simple way to capture the relative populations of the conformations. As illustrated in Figure 3, the two local free energy minima occupied by the two conformations correspond to two populated states denoted inactive (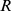) and active (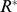). In the absence of a ligand, 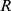 is the most populated state (pale green); at a saturating ligand concentration, the 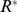 state (orange) becomes the more populated state, indicating a population shift from 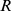 to 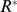 following ligand binding. Given the equilibrium constant, 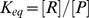 for the reaction 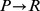, an exact measurement of the relative populations of *P* versus *R* can be expressed by Gibbs free energy 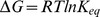, where *R* is the ideal gas constant and 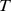 the temperature. In line with the ATSM, 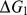 and 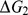 in Figure 5A are the free energy differences between the 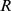 and 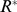 states, respectively for prior (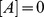) and following (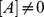) binding. Using the equilibrium equations defined in Figure 2A, we obtain 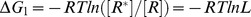 and 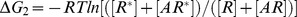. When ligand *A* approaches its saturation concentration (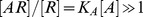 for 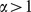 or 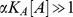 for 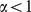), the free energy difference, 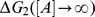, can be approximated by 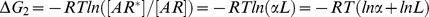. Then, the overall allosteric population shift due to ligand binding, expressed by the free energy change, is given by 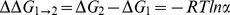. In terms of the free energy landscape, the 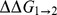 can be alternatively expressed by two components: 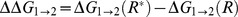, where 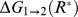 refers to a favorable stabilization energy with respect to the active 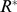 conformation through ligand binding, and 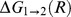 to destabilization energy with respect to the inactive *R* conformation. In addition to Figure 5A with 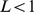, to further clarify the relationship of 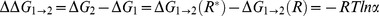, Figure S1 in the Supporting Information provides a free energy landscape with 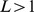.

## Structural View of Allostery: One Allosteric Site with One Functional Site Coupled by *α*

With a given *L* in the ATSM, the maximal extent of allosteric activation 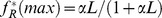 (Figure 2B) depends solely on the parameter 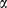. The extent of the population shift following binding, with a maximum free energy change of 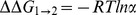, also indicates dependence on 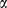. The population shift in a quantitative free energy landscape, already a simplified thermodynamic view, can also simplify the structural view of allostery, as shown in Figure 5B for two structural sites linked by an allosteric coupling constant 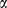. In terms of binding reversibility at both sites, the double arrow indicates that positive 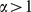 (or negative 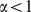) cooperative binding is bidirectional; binding at the functional site will increase (decrease) binding affinity at the allosteric site. The coupling constant 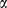 determines the population shift of the active conformation due to binding at the allosteric site. The traditional structural view explains allostery by (1) the coupling between the two distal sites, which is described as strain energy created at the allosteric site, and releasing its energy through a propagation pathway toward the functional site with a consequent shift of the population; and (2) the highly correlated (or concerted) motion through a set of interacting residues which link the sites. Both explanations imply higher allosteric efficacy with a stronger coupling. However, the coupling through the propagation channel does not determine the allosteric efficacy; it is merely a necessary condition for allostery. What determines the allosteric efficacy is the sum of the extent of stabilization of the active conformation plus the destabilization of the inactive conformation following ligand binding.

## One Allosteric Site with Two (Independent) Functional Sites

Biased agonist binding implies that GPCRs can adopt multiple active states [56]. The structural view of the ATSM with one allosteric site and one functional site can be expanded to a system with one allosteric and two functional sites if the population of the two distinct active sites are regulated independently by the allosteric site. This argues that the two functional conformations are not mutually exclusive. Instead, they coexist and the total concentration of 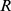 is independent for both active conformational states with 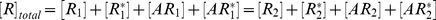. Figure S2 gives the equilibrium cycles for the two functional states with one ligand. The structural view with one allosteric site for two independent activities is given in Figure S3, showing two independent allosteric efficacies, *α1* and *α2*, for two independent functional sites.

## ATSM of Two Allosteric Sites with One Functional Site

**Figure 6:**
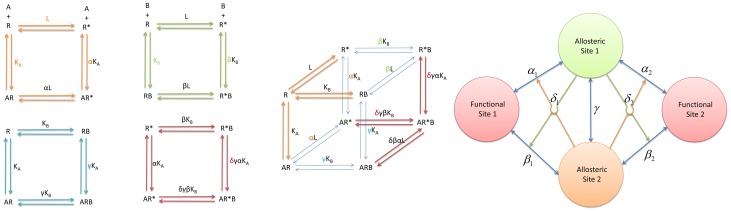
The extended ATSM with two allosteric ligands. (A) The model has ten species related by four equilibrium cycles with seven parameters. The first equilibrium cycle (orange) specified by the 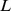, 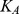, and 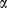 is exactly the same as in the simplest ATSM (Figure 2A), giving 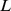 the equilibrium constant between the two states, 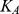 the binding affinity of ligand 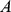 bound to inactive 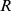, and 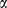 the allosteric intrinsic efficacy of ligand 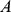. The second equilibrium cycle 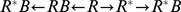 (pale green) describes the second ligand binding similar to the first ligand binding, assigning 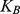 and 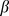 respectively as the binding affinity and the allosteric intrinsic efficacy of ligand 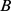. In the third equilibrium cycle 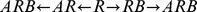 (cyan), the sixth parameter 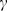 administers the binding cooperativity between ligand 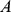 and 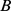 upon the formation of the ternary complex 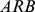. Similarly, the seventh parameter 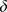 governs the activation cooperativity between ligand 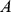 and 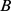 through the formation of 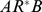 in the fourth equilibrium cycle 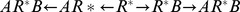 (red). (B) The complete equilibrium cycles of the extended ATSM. The four essential equilibrium cycles of the extended ATSM in (A) are combined into a cubic shape of a complete cycle. To guide the visualization, the two corners of the complete cycle are highlighted by colored equilibrium arrows for species 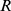 and 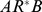 and colored parameters for referencing back to the individual essential equilibrium cycle. (C) The structural view of allostery with two allosteric site and two (independent) functional sites. The drawing is based on two assumptions. First, the populations of the two functional sites are regulated independently by two distinct allosteric sites. Second, the two functional conformations coexist. The allosteric coupling set (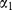, 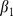, and 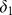) for functional site 1 and a duplicated set of independent allosteric efficacies (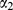, 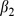, and 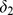) for functional site 2 are similar to the description in Figure 6B. These two sets of coupling are linked by a shared binding cooperativity *γ*, coupling the two allosteric sites.

The discovery of allosteric modulators [35], [57]–[59] of GPCRs and even ternary synergistic allostery [60] requires an extension of the simplest allosteric two-state model to accommodate two or more allosteric ligands bound at distinct sites. Indeed, such an extension has been formulated by Hall [61]. The extended ATSM gives five more species including ligand 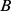, two binary complexes (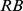 and 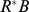 for 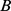 bound to inactive and active 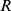), and two ternary complexes (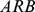 and 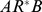), with four additional parameters (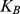, 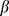, 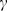, and 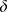, defined below). We refer to Hall's model as the extended ATSM with two allosteric ligands. Below, we only describe briefly the extended ATSM and refer the reader to the original paper [61]. In Figure 6A, ten species are related by four equilibrium cycles with seven parameters. In the first cycle (orange), the 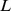, 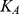, and 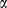 in the extended ATSM are the same as for the single ligand ATSM, with *L* the equilibrium constant between the two states, 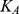 the binding affinity of ligand 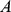 bound to inactive 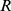, and 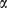 the allosteric intrinsic efficacy of ligand 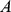. Similarly, the second cycle 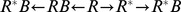 (pale green) defines 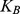 and 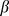 respectively as the binding affinity and the allosteric intrinsic efficacy of ligand 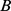. As revealed in the third cycle 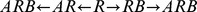 (cyan), 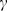 provides the binding cooperativity between ligands 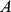 and 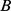 via formation of ternary complex 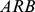. The parameter 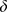 governs the cooperativity in the activation by ligands 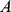 and 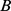 through formation of 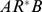 as indicated in the fourth equilibrium cycle 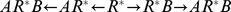 (red). The cubic shape of the cycles in the extended ATSM is given in Figure 6B.

As in the simplest ATSM, the free energy differences between the *R* and R* states in the extended ATSM are defined as 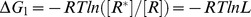 and 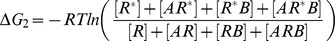, respectively for prior (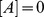 and 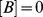) and following ([A]≠0 and [B]≠0) binding. Again, as both ligands approach their saturating concentrations, 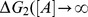, 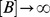) can be approximated by 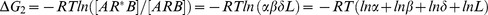. The population shift due to dual ligand binding as expressed by the free energy change becomes 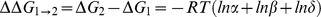. The energy landscape of the extended ATSM is given in Figure S4. Overall, the free energy change for the population shift including the contribution from the efficacy of the second ligand is 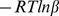 and the activation cooperativity 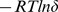. The corresponding structural view is in Figure 5C, with the allosteric efficacy 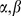 reflecting independent communication between the two allosteric sites and the functional site. The communication between the two allosteric sites expressed by the binding cooperativity 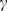 does not affect the allosteric efficacy. In contrast, the activation cooperativity 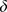 provides the sum of the allosteric effects from the coupling between allosteric site 1 and the allosteric coupling 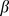 plus from allosteric site 2 and allosteric coupling 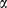. As in the simpler ATSM, communications between the sites are through existing channels and ligand binding determines the allosteric efficacy and the activation cooperativity.

## Two Allosteric Sites with Two (Independent) Functional Sites

Two complex extensions of ATSM have been developed [62]. The discovery of biased allosteric modulators [31] of GPCRs provides a simple extension of the structural view, similar to the case of one allosteric site and two (independent) functional sites. If the populations of the two functional sites are regulated independently by two distinct allosteric sites, and the two functional conformations can coexist, the structural view can be described by two allosteric sites and two (independent) functional sites, as in Figure 6C. The allosteric coupling set (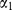, 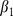, and 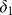) for functional site 1 is added to a duplicated set of independent allosteric efficacies (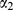, 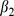, and 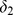) for functional site 2, with the shared binding cooperativity *γ* linking the two allosteric sites.

## Agonist Classification

The simplified structural view of allostery with experimentally measureable parameters can usefully define agonist types. The classifications are summarized in Table 1. The system is specified in column 1, with the total number of allosteric and functional sites as defined by the structural view of allostery. A1 and A2 in column 1 correspond to the presence of one or two allosteric sites, and F1 and F2 to one or two functional sites. The first four classifications of the A1/F1 system (one allosteric and one functional site) are given in Figure 4 as full agonist, partial agonist, natural antagonist, and inverse agonist. In the rest of the classification, the four types of couplings between allosteric and functional sites are sorted into only two categories: agonist for 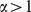 and antagonist for 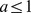. Two couplings in a system containing a single allosteric and two independent functional sites (A1/F2), *α_1_* and *α_2_*, are in the Supporting Information (Figure S3). If both couplings are not in the same agonist category, it is classified as biased agonist. Otherwise it is classified as an agonist (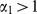 and 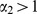) or antagonist (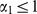 and 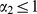). In the A2/F1 system of Figure 5C, the positive (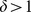) or negative (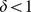) modulator is defined with 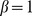, which means no direct coupling between the modulator and functional sites. Similarly, the A2/F2 system there can be defined as biased agonist with β_1_ and β_2_ not in the same agonist category and biased modulator with 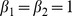 and 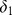, 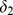 not in the same agonist category.

**Table 1:** Agonist classification by the simplified structural view of allostery.

System	Allosteric site 1	Allosteric site 2	α_1_	β_1_	δ_1_	α_2_	β_2_	δ_2_
A1/F1	Full agonist		αL≫1					
A1/F1	Partial agonist		α>1					
A1/F1	Natural antagonist		α = 1					
A1/F1	Inverse agonist		α<1					
A1/F2	Agonist		α_1_>1			α_2_>1		
A1/F2	Antagonist		α_1_≤1			α_2_≤1		
A1/F2	Biased agonist		α_1_>1			α_2_≤1		
A2/F1	Agonist	Positive modulator	α>1	β = 1	δ>1			
A2/F1	Agonist	Negative modulator	α>1	β = 1	δ<1			
A2/F2	Agonist	Agonist	α_1_>1	β_1_>1	δ_1_>1	α_2_>1	β_2_>1	δ_2_>1
A2/F2	Agonist	Biased agonist	α_1_>1	β_1_>1	δ_1_>1	α_2_>1	β_2_≤1	δ_2_≤1
A2/F2	Agonist	Biased modulator	α_1_>1	β_1_ = 1	δ_1_>1	α_2_>1	β_2_ = 1	δ_2_≤1

Column 1 gives the number of allosteric and functional sites as specified in the structural view of allostery. For example, A1/F2 stands for the allosteric system containing one allosteric site and two functional sites. The second and third columns give the classification of ligand bound at the two allosteric sites. Blanks in the table mean that the item does not apply to the classification. The classification is based on the next six columns which correspond to the six allosteric efficacies defined in Figure 5B, Figure S3, Figure 5C, and Figure 6C, respectively, for the A1/F1, A1/F2, A2/F1, and A2/F2 systems.

## How Allostery Works from the Thermodynamic, Free Energy Landscape of Population Shift, and Structural Standpoints

Above, we described allostery from three perspectives, thermodynamic, free energy landscape of population shift, and structural, with exactly the same allosteric efficacies emerging from the equations of the allosteric two-state model. Below, we explore how allostery works in each.

The thermodynamic view tells us that allostery works via a population shift from the inactive to the active state (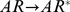), with the ligand preferring to bind the active over the inactive conformation. It is the preferred binding affinity, (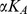 vs. 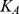) not the overall binding affinity, that puts forth the allosteric effect. Although the binding preference (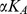) in the ATSM, which captures the conformation selection scheme (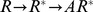), fits the allosteric population shift (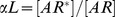), the possibility of an activation path via induced-fit (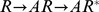) cannot be ruled out. However, as long as the thermodynamic equilibrium is established rapidly, we do not concern ourselves with which path is preferred to reach the activation state. The free energy landscape of the ATSM also tells us that allostery works via population shift from the inactive to the active state with 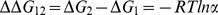. This indicates that allostery can be quantified by allosteric efficacy (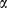) and confirms the implicit inference from the thermodynamic view. The more surprising implication results from expressing 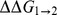 by two components, 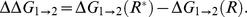 The first component 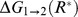, dubbed stabilization energy, specifies the amount of the active 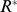 conformation being stabilized by the binding event; the second component 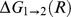, dubbed destabilization energy, corresponds the amount of the inactive 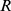 conformation being destabilized through the ligand binding. When both the agonist-bound active structure and the inactive structure are known, these two energy terms can solve the fundamental underpinnings of allostery.

According to the structural view, the allosteric coupling between two sites does not involve ligand binding, which is the trigger of allostery. So, how does allosteric coupling between two sites help to explain allostery? First, coupling implies that the conformation at the allosteric site must to some degree be correlated with the conformation at the functional site. When the conformations at the functional site breathe dynamically between the two free energy basins of inactive and active states, conformational changes take place at the allosteric site. Even slight structural differences at the allosteric site can be sufficient to set the stage for a binding event, via correlated modulation of the population of the activated state. Second, the coupling also implies that certain key interactions are responsible for structural stability in only one state, active or inactive, but not both states. These shape the conformational change at the allosteric site.

Finally, we note that the implications of our unified model are mostly consistent with those of the recent EAM model [14], [17]; however, they differ in the key conceptual guideline for determining agonism. Our model emphasizes that it is the specific interactions between the ligand and the host protein, dubbed as the stabilization of the active state and destabilization of the inactive state, that determine the allosteric efficacy and agonism in a ligand binding event. In contrast, the EAM states that the allosteric mechanism is robustly encoded in the ensemble, and does not require different interactions for a ligand acting as an agonist versus as an antagonist [17]. The conclusion that ligand-specific interactions can be disregarded is not surprising, since the EAM model *a priori* assumes implicit fixed interactions between the ligand and the host. However, the energy terms defined in the EAM can be reformulated within the framework of the ATSM [36] to express allosteric efficacy; that is, the EAM can be considered as a yet another, different formulation of the ATSM. Thus, we believe that the implications of the EAM may have stemmed from the underlying premise of overlooking changes in the interactions between the ligand—agonist or antagonist—and the host, which in the EAM model were implicitly expressed by the distinct set of energies.

## Conclusions

Our purpose in writing this Perspective is to survey points of view on allostery and synthesize them via a mathematical model to obtain a coherent understanding of the question of how allostery works. Starting with the allosteric two-state model, we link the thermodynamic model of allostery and the free energy landscape of population shift. This not only unifies allosteric models based on the same allosteric descriptors; it also provides a simplified structural view of allostery and a set of measureable parameters which allow distinguishing among agonist, biased agonist, modulator, and biased modulator. The emerging new unified view accommodates the three basic elements of allostery: the thermodynamics point of view, the free energy landscape of the population shift, and the structural point of view.

The ***thermodynamic view*** of the allosteric two-state model provides experimentally measurable parameters, which emphasize that allostery reflects preferred ligand binding to one of the two (active, inactive) states. The ***free energy landscape*** representation incorporates the preferred ligand binding formulation and transforms the conceptual framework of population shift into an amount of energy which is proportional to the stabilization of the active conformation and (or) destabilization of the inactive conformation, in the case of allosteric activation. The integrated view translates these into an important rule for the classical ***structural view of allostery***: by itself, the structural coupling (or the propagation pathway) between the functional (active) and the allosteric sites does not involve allostery; it merely identifies high correlation between the conformations of the two sites. The importance of this rule can be assessed by the number of publications using couplings and propagation pathways to delineate allostery.

Beyond generalities, an important question is *how does allostery work in a specific system of interest*? To decipher the allosteric mechanism in a given protein one needs three elements: first, the active conformation which is responsible for the specific function. Structures are essential in order to understand how the agonists act as allosteric triggers. This conformation should populate one of the local free energy basins (the “active basin”) at the bottom of the folding funnel. This emphasizes that an allosteric event such as ligand binding does not create a new conformational state; it only shifts the population among existing states. Second, one needs a set of interacting residues which make up the allosteric communication pathway between the active and allosteric sites; this implies that evolution has set the relative populations of the active versus inactive conformations and linked them to critical pathway residues. Mutations of those residues—if not involving both interacting partners—will affect the allosteric propagation and the population of the active conformation. Knowledge of these mutations is useful because they can be explored by methods such as fast ensemble sampling [46] or covariance analysis of NMR chemical shifts [53]. Third, to figure out an activation event, we suggest considering whether ligand binding at the allosteric site stabilizes the active conformation or destabilizes the inactive conformation or both at the same time [1]. Collectively, we expect that the unified view of “how allostery works” will help guide allosteric drug discovery and provide structural insight into multiple signaling pathways mediated by biased agonists. Finally, the unified view helps unravel the structural mechanism of allostery and advances the notion that allosteric switches in cellular circuits, which are governed by optimized active and inactive conformations with dominant populations, co-evolved with their associated allosteric ligands.

## Supporting Information

Figure S1
**The free energy landscape of ATSM.** Instead of the case of 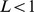 in Figure 5A, the drawing is based on the case of 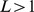 for a clear visualization of the relationship of 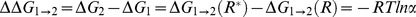.(TIF)

Figure S2
**Equilibrium cycles for two functional states with a single ligand.** Two assumptions have been made for the drawing. First, the population of the two distinct active states are regulated independently by the ligand. Second, the total concentration of 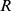 is independent of both active conformational states with 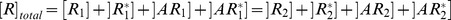. The descriptions of each equilibrium cycle are similar to those described in Figure 2A.(TIF)

Figure S3
**The structural view of allostery for one allosteric site with two independent functional sites.** The drawing shows one allosteric site is independently coupled to two functional sites with allosteric efficacies, 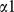 and 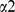, respectively. The description in Figure 5B should also apply to individual coupling here.(TIF)

Figure S4
**The free energy landscape of the extended ATSM.** The energy landscape drawing is similar to Figure 5A for ATSM with two changes. First, instead of single ligand binding, here there are dual binding events with ligand A and ligand B. Second, the free energy change responsible for population shift includes additional contribution from the intrinsic efficacy of the second ligand, 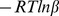, and the activation cooperativity, 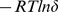, with 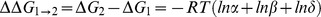.(TIF)
