# Supplementary figures and images for: A Unified View of “How Allostery Works”
Source: PLoS Comput Biol. 2014 Feb 6;10(2):e1003394. doi: 10.1371/journal.pcbi.1003394 (PMC3916236; doi:10.1371/journal.pcbi.1003394)

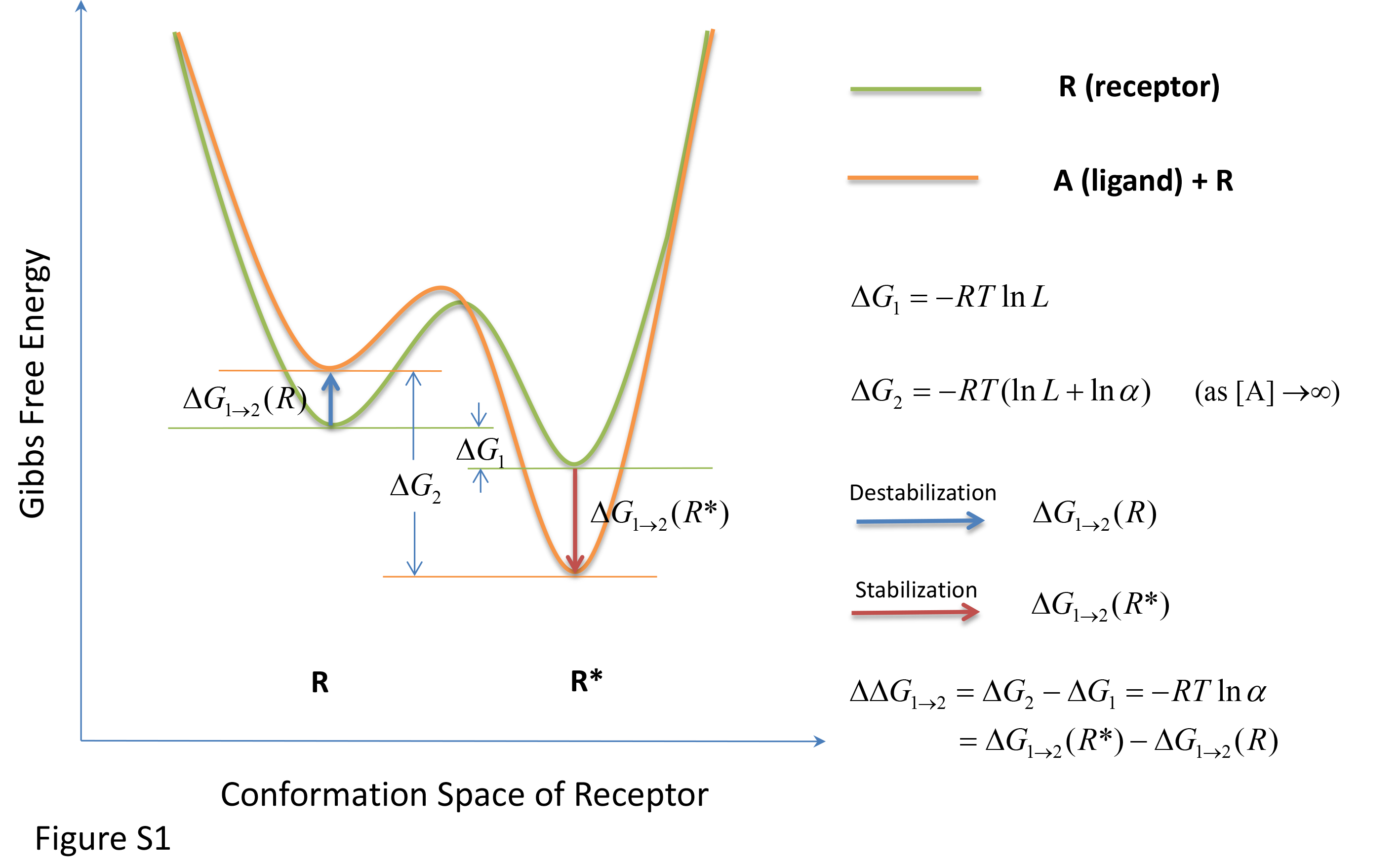

Supplement: Figure S1 — The free energy landscape of ATSM. Instead of the case of in Figure 5A, the drawing is based on the case of for a clear visualization of the relationship of . (TIF) [file pcbi.1003394.s001.tif]

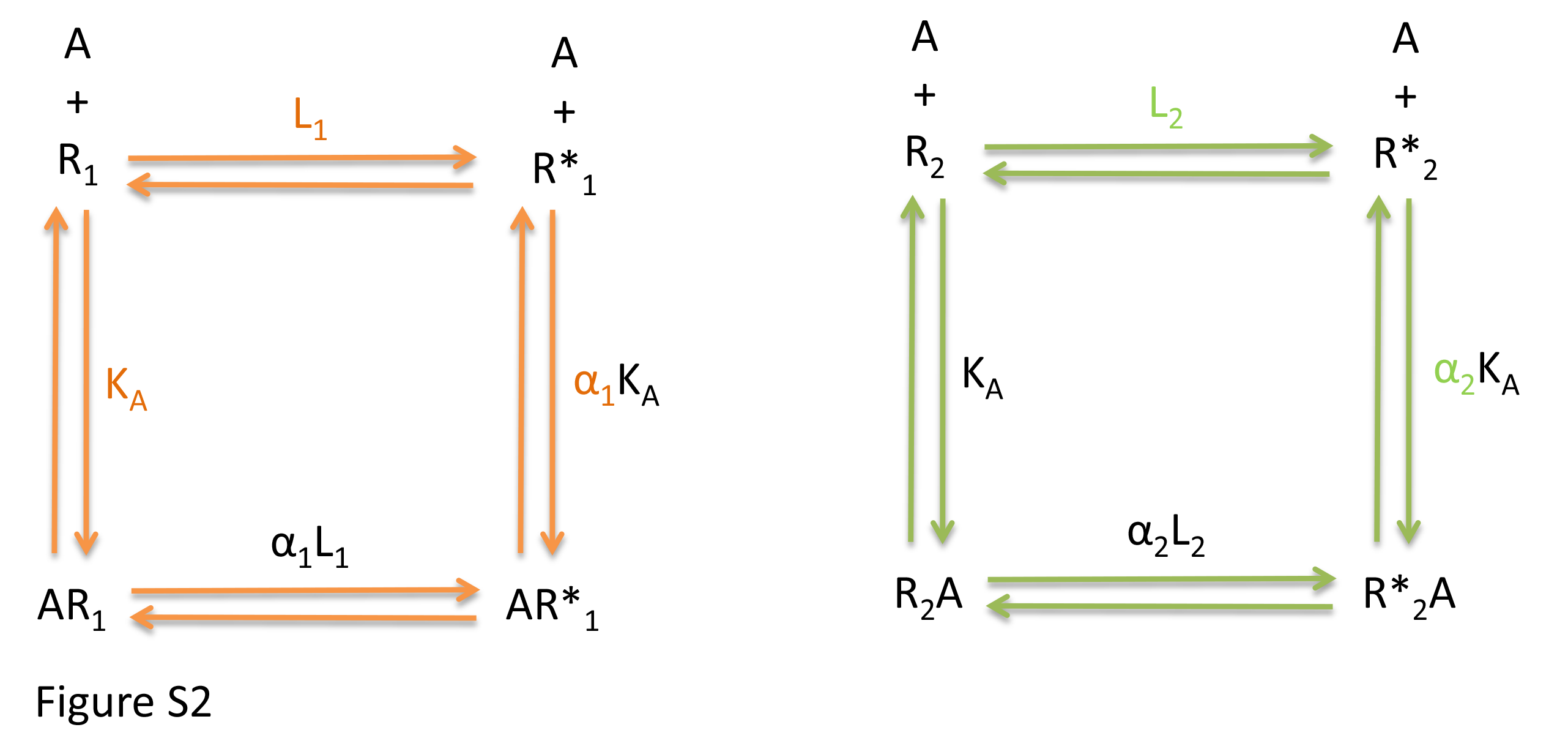

Supplement: Figure S2 — Equilibrium cycles for two functional states with a single ligand. Two assumptions have been made for the drawing. First, the population of the two distinct active states are regulated independently by the ligand. Second, the total concentration of is independent of both active conformational states with . The descriptions of each equilibrium cycle are similar to those described in Figure 2A. (TIF) [file pcbi.1003394.s002.tif]

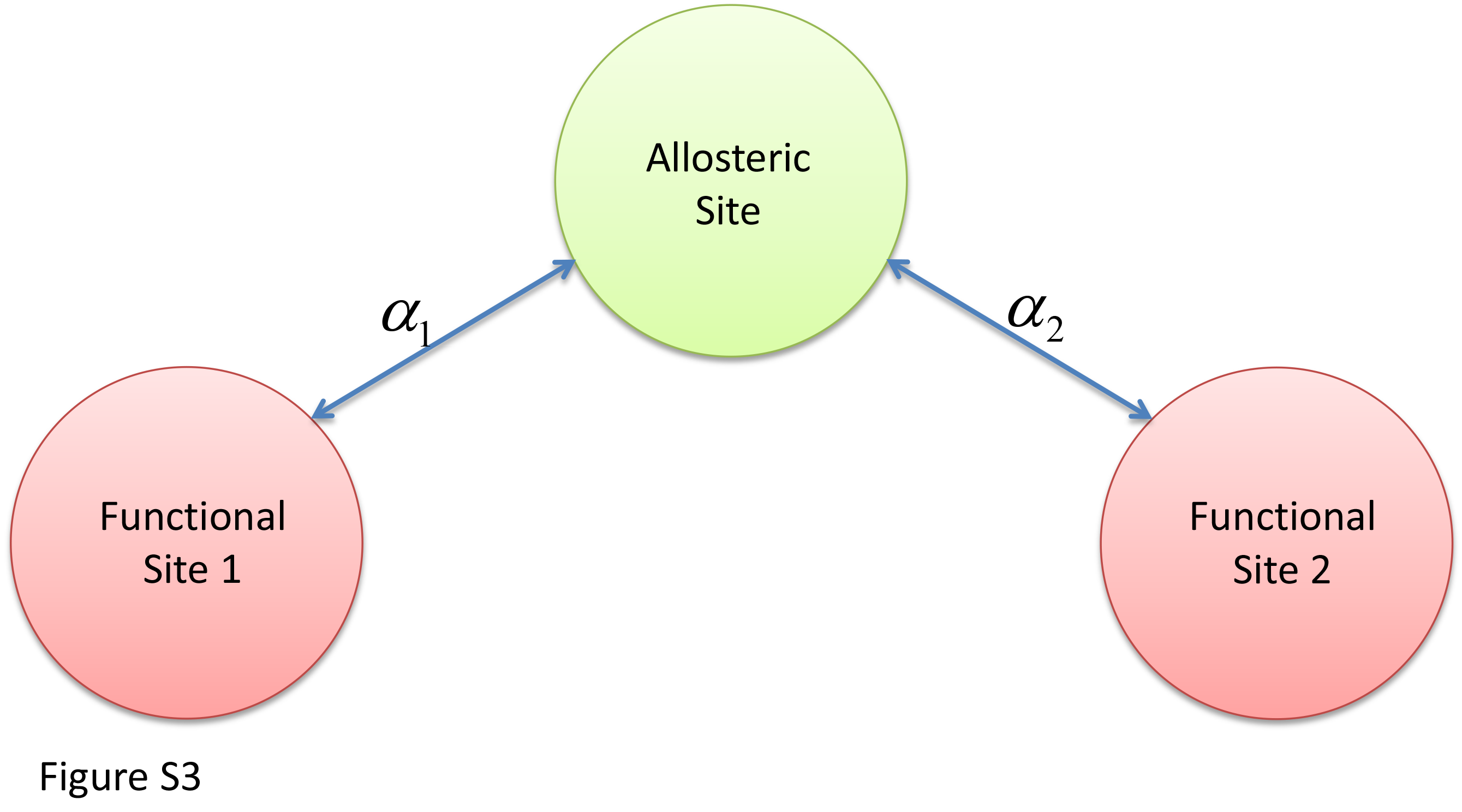

Supplement: Figure S3 — The structural view of allostery for one allosteric site with two independent functional sites. The drawing shows one allosteric site is independently coupled to two functional sites with allosteric efficacies, and , respectively. The description in Figure 5B should also apply to individual coupling here. (TIF) [file pcbi.1003394.s003.tif]

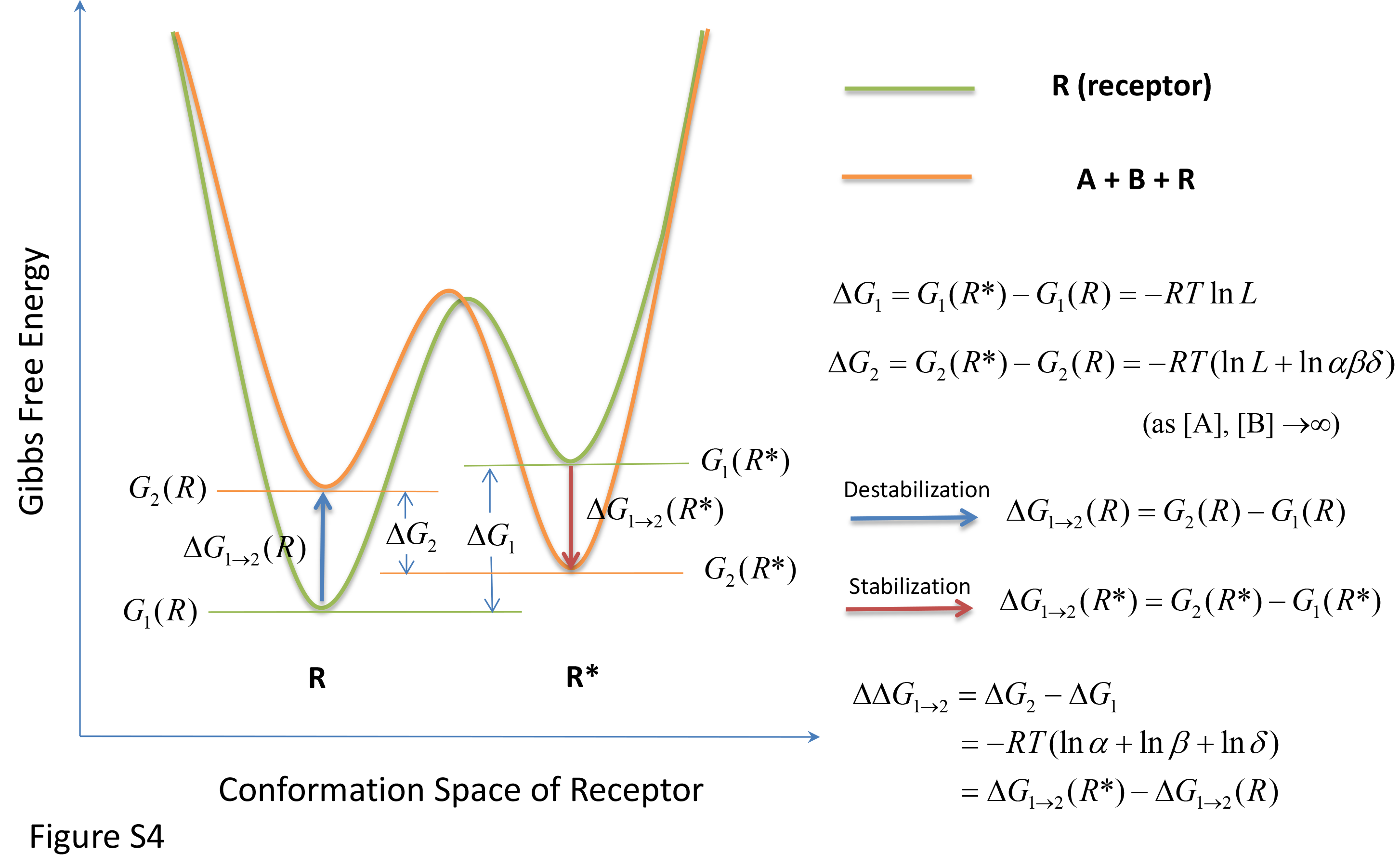

Supplement: Figure S4 — The free energy landscape of the extended ATSM. The energy landscape drawing is similar to Figure 5A for ATSM with two changes. First, instead of single ligand binding, here there are dual binding events with ligand A and ligand B. Second, the free energy change responsible for population shift includes additional contribution from the intrinsic efficacy of the second ligand, , and the activation cooperativity, , with . (TIF) [file pcbi.1003394.s004.tif]
